# Lower re‐revision rates after UKA‐to‐TKA than UKA‐to‐UKA revision for failed medial unicompartmental knee arthroplasty at mid‐term follow‐up

**DOI:** 10.1002/jeo2.70778

**Published:** 2026-05-25

**Authors:** Kevin‐Arno Koch, Jakob Freytag, Mustafa Hariri, Raphael Trefzer, Paul Mick, Andre Lunz, Tilman Walker, Johannes Weishorn

**Affiliations:** ^1^ Department of Orthopaedic Surgery University Hospital of Heidelberg, Schlierbacher Landstrasse 200a Heidelberg Germany

**Keywords:** knee replacement arthroplasty, reoperation, treatment failure, treatment outcome, unicompartmental knee arthroplasty, patient reported outcome measures

## Abstract

**Purpose:**

Revision following unicompartmental knee arthroplasty (UKA) remains challenging, and optimal revision strategy is still debated. This study analysed failure mechanisms after aseptic medial UKA revision and compared mid‐term outcomes between UKA‐to‐UKA and UKA‐to‐total knee arthroplasty (TKA) revision procedures.

**Methods:**

A retrospective single‐centre study included 113 consecutive aseptic UKA revisions performed between 2001 and 2020. Patients were stratified into UKA‐to‐UKA (*n* = 38) and UKA‐to‐TKA (*n* = 75) groups. Progression of lateral osteoarthritis (31%) and aseptic loosening (27%) were the most common indications for revision. Re‐revision rates were evaluated and implant survival was assessed using Kaplan–Meier analysis with log‐rank testing and Cox regression. Functional outcomes were evaluated using validated patient‐reported outcome measures (Oxford Knee Score, Knee Society Scores, UCLA activity score, visual analogue scale) and range of motion.

**Results:**

During follow‐up, re‐revision occurred in 8 of 38 UKA‐to‐UKA revisions (21.1%) and in 3 of 75 UKA‐to‐TKA revisions (4.0%). Re‐revision after UKA‐to‐TKA revision occurred due to suspected early infection (*n* = 2) and aseptic loosening (*n* = 1), whereas after UKA‐to‐UKA revision the main causes were aseptic loosening (*n* = 3), unexplained pain (*n* = 3) and bearing dislocation (*n* = 2). Overall, 10‐year implant survival was 89.7%. Implant survival at 10 years was higher following UKA‐to‐TKA revision compared with UKA‐to‐UKA revision (95.3% vs. 78.6%; log‐rank *p* = 0.003). PROMs did not differ significantly between groups, whereas range of motion was greater after UKA‐to‐UKA revision (126° vs. 114°, *p* < 0.001).

**Conclusion:**

Conversion of medial UKA to TKA was associated with lower re‐revision rates compared with UKA‐to‐UKA revision, while patient‐reported outcomes were comparable between strategies. UKA‐to‐UKA revision may remain appropriate in selected cases with surgically addressable failure mechanisms. These findings support an individualised revision strategy based on the underlying cause of failure.

**Level of Evidence:**

Level III, retrospective cohort study.

**Abbreviations**
AKSS‐F: functional American Knee Society Score
AKSS‐O: objective American Knee Society Score
BMI: body mass index
CI: confidence interval
CR: cruciate retaining
DAIR: debridement, antibiotics and implant retention
FU: follow‐up
HR: hazard ratio
OKS: Oxford knee score
OR: odds ratio
PASS: patient acceptable symptom state
PROMs: patient‐reported outcome measures
PS: posterior stabilised
RH: rotating‐hinge
ROM: range of motion
SC: semi‐constrained
SD: standard deviation
TKA: total knee arthroplasty
UCLA: University of California Los Angeles Activity Score
UKA: unicompartmental knee arthroplasty
VAS: visual analogue scale

## INTRODUCTION

Unicompartmental knee arthroplasty (UKA) offers several advantages over total knee arthroplasty (TKA), including reduced soft tissue trauma, preservation of native joint kinematics, faster recovery and lower perioperative morbidity in appropriately selected patients [1, 10, 17, 38]. Advances in implant design and surgical technique have improved survivorship, particularly in high‐volume settings [3, 15, 29, 40]. Nevertheless, registry data consistently demonstrate that UKA remains associated with a two‐ to threefold higher revision risk compared to TKA, with only modest improvements over the past decade [5, 20, 26, 27]. As the utilisation of UKA continues to rise, the absolute number of revision procedures is increasing accordingly, underscoring the importance of defining optimal revision strategies [18, 35, 36, 39].

The literature on revision UKA has expanded substantially in recent years. Most contemporary studies focus on UKA‐to‐TKA conversions [7, 13, 19, 21, 28, 30, 32], whereas data on UKA‐to‐UKA revision remain scarce [4, 22, 31]. Recent series by Pumford et al. and Ibach et al. demonstrate favourable survivorship and functional performance following UKA‐to‐TKA revision, supporting conversion as a reliable treatment option in many cases [13, 30]. In contrast, Restrepo et al. recently analysed both UKA‐to‐UKA and UKA‐to‐TKA revisions and observed higher early failure rates in the UKA‐to‐UKA group [31]. Importantly, the majority of UKA‐to‐UKA procedures in that cohort were performed for acute periprosthetic joint infection (PJI) and treated with implant retention, follow‐up focused predominantly on short‐term outcomes and functional results were not reported. These factors may substantially influence re‐revision risk and limit extrapolation to aseptic mechanical failure patterns.

Furthermore, published series combine septic and aseptic failures or include heterogeneous implant designs and revision indications, complicating interpretation of failure mechanisms and survivorship [4, 31]. Robust mid‐term data in strictly aseptic cohorts remain limited. In addition, UKA‐to‐UKA revision represents a heterogeneous entity that has not been systematically analysed in detail.

Therefore, the present study aims to analyse the causes and characteristics of aseptic revisions following medial mobile‐bearing UKA and to compare mid‐term outcomes including re‐revision rates and patient‐reported functional outcomes after UKA‐to‐UKA revision and UKA‐to‐TKA revision within a single high‐volume tertiary referral centre. We hypothesised that UKA‐to‐TKA revision may be associated with improved implant survivorship, while functional outcomes between strategies would be comparable.

## MATERIALS AND METHODS

### Study design and patient selection

In this single‐centre retrospective study, all patients who underwent aseptic revision surgery following medial UKA at a tertiary referral centre between 2001 and 2020 were identified through an institutional review board–approved database. The study was conducted in accordance with the Helsinki Declaration of 1975, as revised in 2018, and institutional review board approval was obtained. Written informed consent was obtained from all participating patients prior to inclusion.

During the study period, a total of 2462 primary unicompartmental knee arthroplasties were implanted at our institution. In the same timeframe, 151 UKA revision procedures were performed, including both internally implanted and externally referred cases.

Patients were included if they had received a medial UKA using the Oxford partial knee implant (Zimmer Biomet Inc.) and underwent aseptic revision surgery. Patients with a history of knee infection or revision due to PJI were excluded to avoid infection‐related confounding. Revisions following PJI have been analysed separately in a previously published study [18]. Cases involving a different primary implant system were also excluded to maintain implant homogeneity.

Of the 151 UKA revisions identified, 132 involved medial UKA and 19 lateral UKA revisions. Twelve medial UKA revisions performed for PJI and six cases involving non‐Oxford primary implant systems were excluded. After applying the inclusion and exclusion criteria, 114 patients remained eligible for analysis. One patient was lost to follow‐up, resulting in a final cohort of 113 patients.

Of these, 38 patients underwent UKA‐to‐UKA revision, while 75 patients were converted to TKA. Patient demographics and perioperative characteristics are presented in Table 1.

**Table 1:** Demographics and perioperative characteristics of patients undergoing UKA‐to‐UKA or UKA‐to‐TKA revision.

Characteristic	UKA‐to‐UKA	UKA‐to‐TKA	*p*‐value
Number of patients, *n*	38	75	
Age at revision, years (range)	64 (45–82)	69 (40–85)	0.02
Sex, *n* (%)			0.46
Men	20 (52.6%)	34 (45.3%)	
Women	18 (47.4%)	41 (54.7%)	
BMI at revision, kg/m² (range)	31.1 (22.8–47.3)	31.0 (20.3–47.1)	0.78
Primary surgery location, *n* (%)			0.13
In‐house	34 (89.5%)	58 (77.3%)	
Referred	4 (10.5%)	17 (22.7%)	
Fixation method at primary UKA implantation, *n* (%)			<0.01
Cemented	22 (57.9%)	65 (86.7%)	
Cementless	16 (42.1%)	10 (13.3%)	

Abbreviations: BMI, body mass index; TKA, total knee arthroplasty; UKA, unicompartmental knee arthroplasty.

### Causes of revision surgery

Clinical records, operative reports, radiographic images and registry data were reviewed to determine the indication for revision surgery and perioperative characteristics. The primary indication for revision surgery was determined based on operative reports, radiographic findings and clinical documentation and was classified independently by two investigators. Disagreements were resolved by consensus.

The main reasons for aseptic revision index surgery after medial UKA, with progression of osteoarthritis in the lateral compartment (35 knees, 31%) and aseptic loosening (31 knees, 27%) as predominant causes, are listed in Table 2. All periprosthetic fractures involved the medial tibia, with one case due to adequate trauma.

**Table 2:** Indications for aseptic revision surgery stratified by revision type.

Reason for index revision	All patients (*n* = 113)	UKA‐to‐UKA (*n* = 38)	UKA‐to‐TKA (*n* = 75)
Progression of lateral osteoarthritis	35 (31%)	‐	35 (47%)
Aseptic loosening	31 (27%)	6 (16%)	25 (33%)
Tibial component	25	4	21
Femoral component	5	2	3
Both components	1	0	1
Mechanical complication	12 (11%)	9 (24%)	3 (4%)
Component malposition	3	2	1
Symptomatic osteophytes	3	3	0
Medial instability	2	2	0
Tibial oversizing	1	1	0
Tibial undersizing	1	0	1
Symptomatic impingement	1	0	1
Liner component failure	1	1	0
Bearing dislocation	12 (11%)	11 (29%)	1 (1%)
Periprosthetic fracture	10 (9%)	3 (8%)	7 (10%)
Unexplained pain	6 (5%)	4 (10%)	2 (3%)
Other causes	4 (4%)	3 (8%)	1 (1%)
Bearing breakage	2 (1%)	2 (5%)	‐
Wear	1 (1%)	‐	1 (1%)

Abbreviations: TKA, total knee arthroplasty; UKA, unicompartmental knee arthroplasty.

### Time to index revision surgery

**Figure 1:**
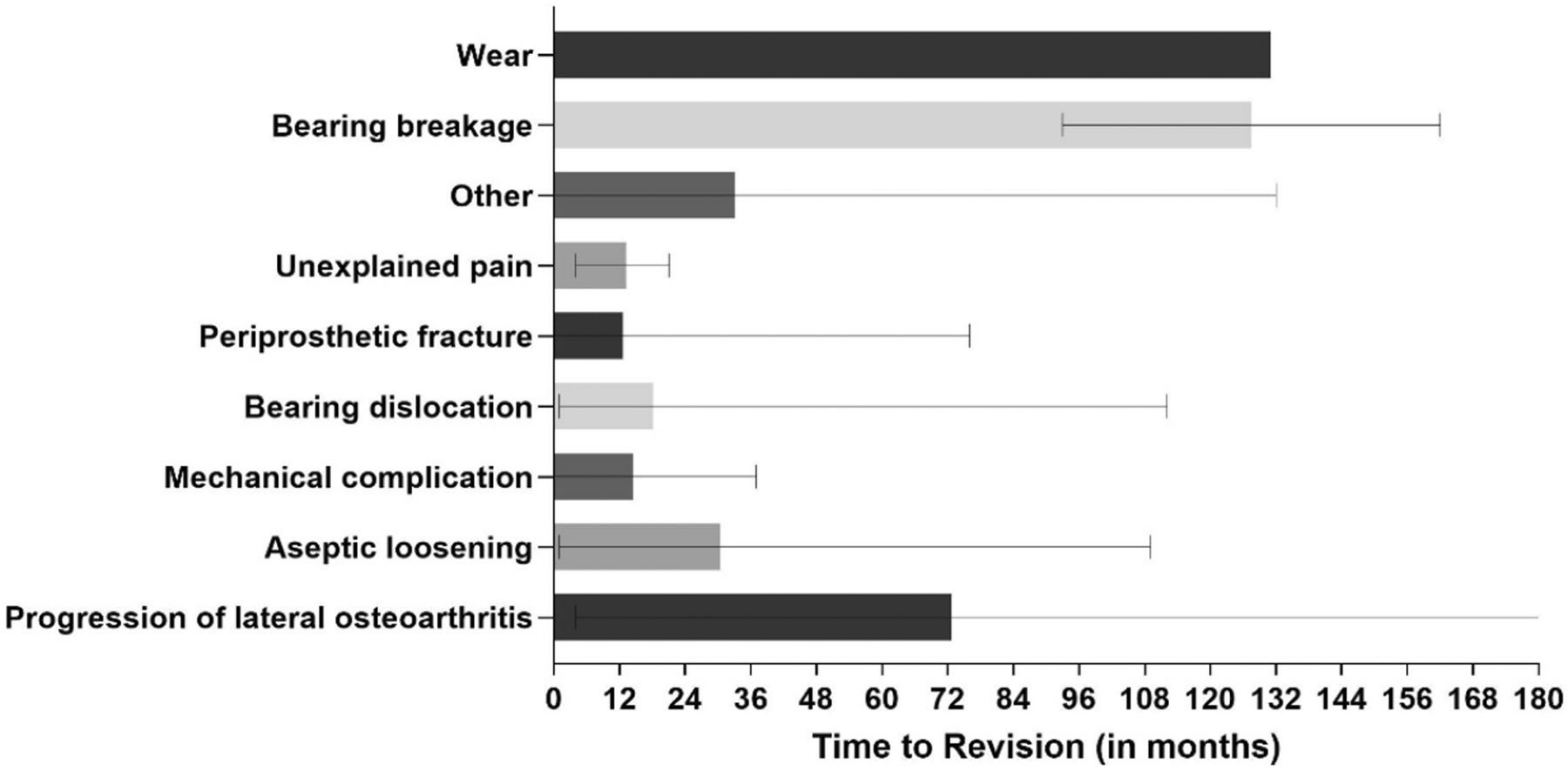
Mean time to revision (with error bars representing range) for each failure mode following medial unicompartmental knee arthroplasty.

Time to index revision was defined as the interval between primary UKA implantation and the aseptic revision procedure. Comparing time to index revision, 54% and 75% of failures occurred within 2 and 5 years, respectively, while only 7% occurred after more than 10 years. The mean time to revision surgery was 40.7 months (range 0–184 months). Mean time to failure for each reason for revision is shown in Figure 1.

### Revision procedures

All primary medial UKA procedures performed in‐house utilised the mobile‐bearing Oxford implant system (Zimmer Biomet Inc.). All revision surgeries were conducted as one‐stage procedures at the same tertiary referral centre.

Prior to revision surgery, all patients underwent standard radiographic imaging and preoperative joint aspiration to exclude PJI. In urgent cases, intraoperative synovial fluid and tissue samples were obtained for microbiological and histological analysis.

As this was a retrospective observational study, treatment allocation was not standardised. The revision strategy was determined at the discretion of the treating surgeon based on clinical assessment, the underlying failure mechanism, bone stock, implant stability and intraoperative findings. In general, UKA‐to‐UKA revision was considered in cases with preserved bone stock and failure mechanisms that were deemed surgically addressable without conversion to TKA, such as isolated mechanical or bearing‐related problems. However, the final decision regarding revision strategy was made intraoperatively according to the surgeon's clinical judgement and operative findings.

A total of 26 surgeons were involved over the study period. All procedures were performed by experienced knee arthroplasty surgeons or under their supervision.

For UKA‐to‐UKA revision, all procedures involved component exchange. At minimum, a liner exchange was performed in every case. In 12 cases, additional partial exchange of the femoral or tibial component was required. Symptomatic osteophytes and intra‐articular loose bodies were removed when indicated.

For UKA‐to‐TKA revision, all procedures involved implant and cement removal, surgical debridement and irrigation. Depending on bone stock and ligament stability, a bicondylar or semi‐constrained TKA was implanted. The predominant implant designs were cruciate‐retaining (*n* = 45) and posterior‐stabilised (*n* = 18), while 11 cases required a semi‐constrained prosthesis and one case involved distal femoral replacement. No revision procedures were performed using robotic assistance.

### Outcomes and clinical review

The primary endpoint of the study was implant survival free from re‐revision for any reason. Re‐revision was defined as any subsequent surgical procedure involving removal or exchange of at least one prosthetic component, including liner exchange. Secondary outcomes included patient‐reported outcome measures (PROMs) and range of motion (ROM).

Clinical and radiographic follow‐up examinations were routinely recommended at 3 months, 1 year, 3 years, 5 years and every 5 years thereafter. Standardised anteroposterior and lateral radiographs obtained during follow‐up were reviewed to assess component position, radiographic signs of loosening and progression of osteoarthritis. However, follow‐up intervals varied in routine clinical practice. For the purpose of this study, patients who had not been evaluated recently were specifically invited for clinical follow‐up examination. At this visit, PROMs and clinical parameters were recorded. The following validated outcome measures were recorded: Oxford Knee score (OKS), American Knee Society Score (AKSS‐O and AKSS‐F), University of California Los Angeles (UCLA) activity score and visual analogue scale (VAS). ROM was measured clinically during last follow‐up visit.

Patients who were unable to attend the examination due to health conditions or other reasons were contacted by mail or phone to provide additional information on complications or revision procedures and to complete the PROMs questionnaires. For deceased patients, information between the last clinical follow‐up and death was obtained using information from relatives, general practitioners and hospital records.

### Statistical analysis

Data were analysed using SPSS Version 27.0 (IBM SPSS Statistics), GraphPad Prism Version 10.0 (GraphPad Software) and R Version 4.3.2 (R Foundation for Statistical Computing). Statistical significance was defined as *p* < 0.05.

Descriptive statistics were calculated as absolute frequencies and means with standard deviations. The UCLA activity score was reported as median with range. Normality of continuous variables was assessed using the Shapiro–Wilk test. Non‐parametric Mann–Whitney *U* tests were used for comparisons between groups.

Implant survival was assessed using the Kaplan–Meier estimator with 95% confidence intervals. Censoring was applied for patients deceased due to unrelated causes, ensuring accurate survival estimation. Based on the sample size, survival was calculated up to 10 years, with a minimum of 24 knees still being at risk. The log‐rank test was used to compare the survival distributions between the groups.

Univariable and multivariable Cox proportional hazards regression analyses were performed to identify potential risk factors for re‐revision. Hazard ratios (HR) with 95% confidence intervals (CIs) were reported. Age and body mass index (BMI) were included as continuous variables, and sex as a categorical variable.

Exploratory subgroup analyses were performed to investigate potential differences in re‐revision risk according to revision timing, revision indication and implant design.

While post‐hoc power calculations indicated adequate power for the observed effect size, such analyses do not overcome the inherent limitations of retrospective survival analyses with unequal group sizes.

## RESULTS

### Study cohort and follow‐up

A total of 113 patients met the inclusion criteria and were included in the final analysis. During follow‐up, 11 patients (9.7%) underwent re‐revision surgery. Of the remaining 102 patients, additional clinical follow‐up data beyond prosthesis status were available for 90 patients, and complete PROMs were obtained in 80 patients.

Twenty‐four patients died during the follow‐up period due to causes unrelated to the implant. These patients were censored at the time of death for survival analysis.

The mean follow‐up duration for the entire cohort was 7.0 years. PROMs were available at a mean follow‐up of 7.5 years (range 1.8–19.9 years).

### Implant survival and re‐revision

During follow‐up, re‐revision occurred in 8 of 38 UKA‐to‐UKA revisions (21.1%) and in 3 of 75 UKA‐to‐TKA revisions (4.0%). The mean time to re‐revision was 16 months (range 0–58 months). Detailed information on individual re‐revision cases is presented in Table 3.

**Table 3:** Details of re‐revision procedures following initial UKA revision.

Case	Time to re‐revision (months)	Reason for index revision	Index procedure (implant type)	Reason for re‐revision	Re‐revision procedure (implant type)
01	0	Periprosthetic fracture	UKA‐to‐TKA (SC)	Suspected early infection	DAIR
02	1	Progression of lateral osteoarthritis	UKA‐to‐TKA (PS)	Suspected early infection	DAIR
03	58	Aseptic loosening	UKA‐to‐TKA (SC)	Aseptic loosening	TKA‐to‐TKA (RH)
01	3	Mechanical complication	UKA‐to‐UKA	Bearing dislocation	UKA‐to‐TKA (PS)
02	6	Unexplained pain	UKA‐to‐UKA	Unexplained pain	UKA‐to‐TKA (CR)
03	7	Bearing dislocation	UKA‐to‐UKA	Bearing dislocation	Two‐stage UKA‐to‐TKA (PS)
04	15	Mechanical complication	UKA‐to‐UKA	Aseptic loosening	UKA‐to‐TKA (SC)
05	18	Unexplained pain	UKA‐to‐UKA	Aseptic loosening	UKA‐to‐TKA (CR)
06	20	Other	UKA‐to‐UKA	Unexplained pain	UKA‐to‐TKA (PS)
07	21	Aseptic loosening	UKA‐to‐UKA	Aseptic loosening	UKA‐to‐TKA (CR)
08	30	Mechanical complication	UKA‐to‐UKA	Unexplained pain	UKA‐to‐TKA (PS)

Abbreviations: CR, cruciate‐retaining; DAIR, debridement, antibiotics and implant retention; PS, posterior‐stabilised; RH, rotating‐hinge; SC, semi‐constrained; TKA, total knee arthroplasty; UKA, unicompartmental knee arthroplasty.

**Figure 2:**
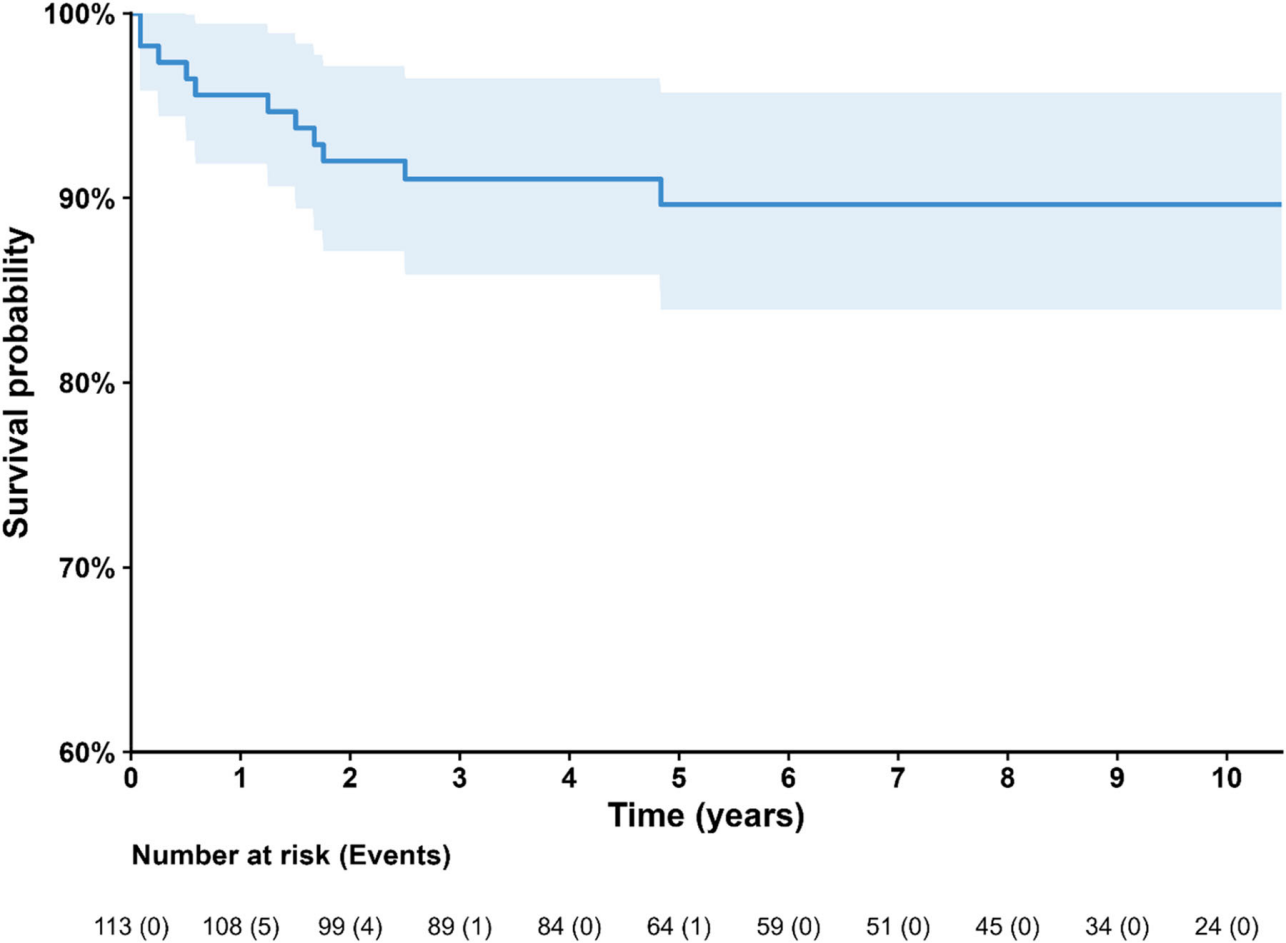
Kaplan–Meier survival curve for all aseptic unicompartmental knee arthroplasty revision procedures (*n* = 113).

Kaplan–Meier survival analysis for all revision procedures demonstrated an implant survival of 95.6% (95% CI 89.7%–98.1%) at 1 year, 92.0% (95% CI 85.2%–95.8%) at 2 years and 89.7% (95% CI 82.0%–94.2%) at both 5 and 10 years (Figure 2).

**Figure 3:**
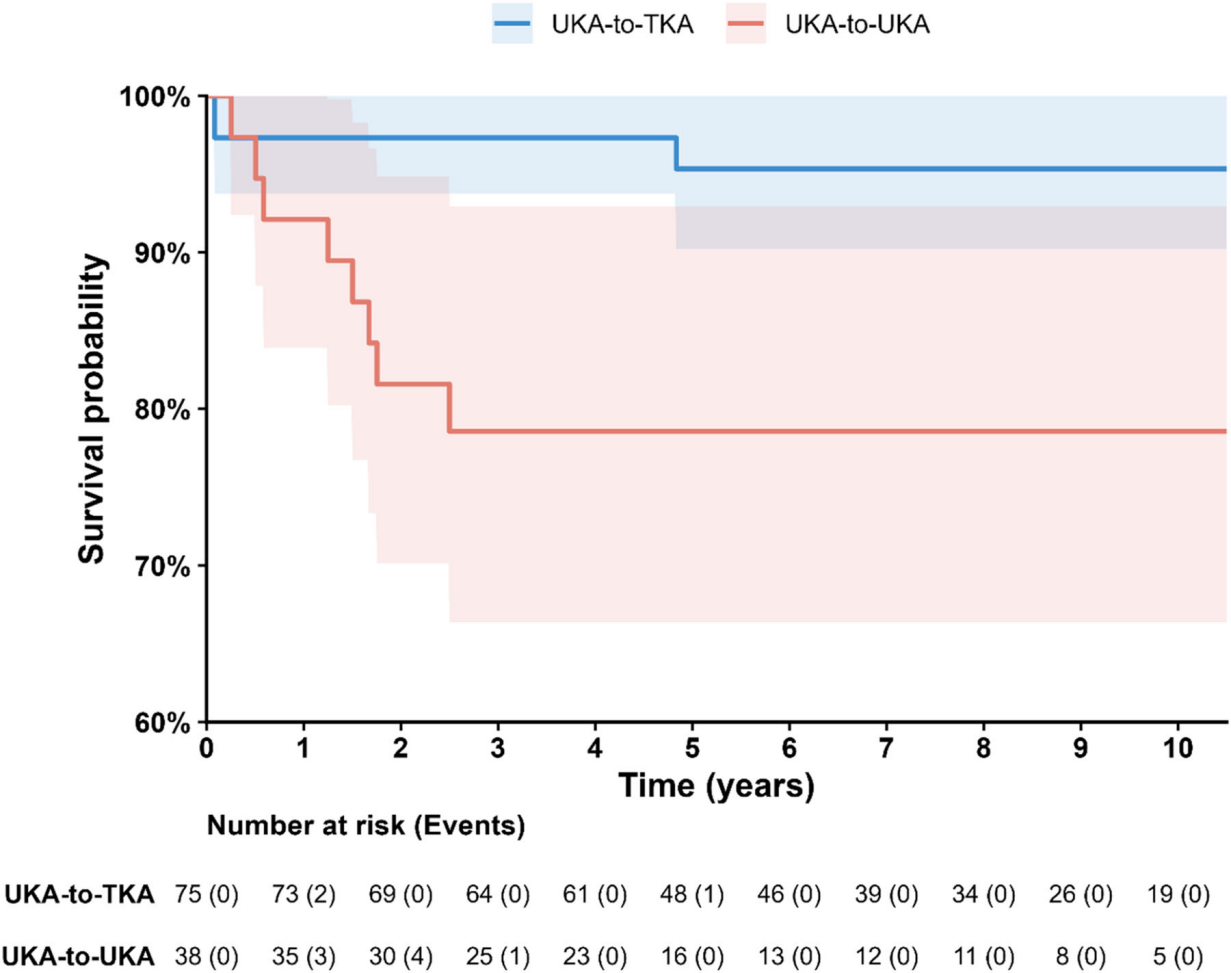
Kaplan–Meier survival curves comparing unicompartmental knee arthroplasty (UKA)‐to‐total knee arthroplasty (TKA) and UKA‐to‐UKA revisions.

When stratified by revision strategy, implant survival at 10 years was significantly higher after UKA‐to‐TKA revision (95.3%, 95% CI 85.9%–98.5%) compared with UKA‐to‐UKA revision (78.6%, 95% CI 61.6%–88.7%) (log‐rank *p* = 0.003) (Figure 3).

In univariable Cox regression analysis, UKA‐to‐UKA revision was associated with a significantly increased risk of re‐revision compared with UKA‐to‐TKA revision. This association remained significant after adjustment for age, sex and BMI (Table 4).

**Table 4:** Univariate and multivariate analysis for comparison of influential factors for UKA‐to‐UKA revision versus UKA‐to‐TKA revision.

Variable	Univariable HR (95% CI)	*p*	Multivariable HR (95% CI)	*p*
UKA‐to‐UKA revision (vs. UKA‐to‐TKA revision)	5.75 (1.52–21.71)	0.010	5.10 (1.32–19.75)	0.018
Sex	‐	‐	1.02 (0.30–3.46)	0.977
Age (per year)	‐	‐	0.98 (0.92–1.04)	0.406
BMI (per kg/m^2^)	‐	‐	1.05 (0.95–1.16)	0.358

Abbreviations: BMI, body mass index; CI, confidence interval; HR, hazard ratio; TKA, total knee arthroplasty; UKA, unicompartmental knee arthroplasty.

### Risk factor and subgroup analyses

Subgroup analyses were performed to explore potential factors associated with re‐revision risk.

Revision performed within 2 years after primary UKA showed a higher re‐revision rate compared with later revision (9/61 vs. 2/52), demonstrating a trend toward reduced implant survival (log‐rank *p *= 0.052; Cox HR 0.25, 95% CI 0.05–1.14; *p* = 0.073).

No significant difference in implant survival was observed between revision periods (2001–2010 vs. 2011–2020; *p* = 0.125).

Within the UKA‐to‐UKA subgroup, higher re‐revision rates were observed for revisions performed for mechanical complications or unexplained pain, whereas revisions for bearing‐related problems, aseptic loosening, or periprosthetic fracture showed lower failure rates. No re‐revisions occurred after revision for periprosthetic fracture.

Isolated liner exchange demonstrated a numerically higher re‐revision rate than component revision, although this difference did not reach statistical significance (*p* = 0.186).

Within the UKA‐to‐TKA subgroup, implant design showed differences in survival. Higher constraint levels were associated with an increased risk of re‐revision (log‐rank *p* = 0.042).

In Cox regression analysis, none of the investigated variables was associated with the risk of re‐revision, irrespective of revision type. Although BMI showed an association with UKA‐to‐TKA revision in the univariable analysis, this was not confirmed after multivariable adjustment (Table 5).

**Table 5:** Univariable and multivariable Cox regression analyses of risk factors for re‐revision, stratified by revision type (UKA‐to‐UKA vs. UKA‐to‐TKA).

Variable	Univariable HR (95% CI)	*p*	Multivariable HR (95% CI)	*p*
UKA‐to‐UKA revision				
Sex	0.63 (0.15–2.64)	0.53	0.63 (0.15–2.70)	0.53
Age (per year)	0.97 (0.90–1.04)	0.41	0.97 (0.89–1.04)	0.37
BMI (per kg/m^2^)	0.99 (0.86–1.12)	0.89	0.98 (0.86–1.13)	0.81
UKA‐to‐TKA revision				
Sex	55.95 (0.01–632,341.31)	0.40	Not estimable^a^	‐
Age (per year)	0.96 (0.86–1.07)	0.46	0.95 (0.82–1.10)	0.47
BMI (per kg/m^2^)	1.23 (1.02–1.48)	0.033	1.26 (0.99–1.59)	0.052

Abbreviations: BMI, body mass index; CI, confidence interval; HR, hazard ratio; TKA, total knee arthroplasty; UKA, unicompartmental knee arthroplasty.

^a^ Hazard ratio not reliably estimable due to complete separation (no UKA‐to‐TKA revisions occurred in male patients).

### Functional outcomes

PROMs were available for 80 patients. Of these, 23 patients underwent UKA‐to‐UKA revision and 57 underwent conversion to TKA. The most common indications for revision among patients with available PROMs were bearing dislocation (*n* = 8) and mechanical complications (*n* = 6) in the UKA‐to‐UKA group, and progression of lateral osteoarthritis (*n* = 30) and aseptic loosening (*n* = 15) in the UKA‐to‐TKA group. The predominant TKA implant design was cruciate‐retaining (*n* = 36) or posterior‐stabilised (*n* = 14).

No significant differences in PROMs were observed between revision strategies (*p* > 0.05) (Table 6). The only significant difference was observed in ROM, which was greater after UKA‐to‐UKA revision compared with UKA‐to‐TKA revision (126° vs. 114°, *p* < 0.001).

**Table 6:** Functional outcomes by revision type at final follow‐up.

PROMs	UKA‐to‐UKA (*n* = 23)	UKA‐to‐TKA (*n* = 57)	*p*‐value
OKS	38.73 (9.25)	36.98 (9.0)	0.29
AKSS‐O	79.47 (18.83)	85.06 (16.83)	0.31
AKSS‐F	79.67 (21.75)	75.42 (26.29)	0.67
UCLA	5 (1‐10)	5 (1‐10)	0.58
VAS	2.64 (2.77)	2.52 (2.84)	0.84
ROM	126 (9.68)	114 (11.47)	<0.001

*Note*: Mean (standard deviation) for Oxford knee score (OKS), American Knee Society Scores (AKSS‐O and AKSS‐F), visual analogue scale (VAS), range of movement (ROM); median (range) score for California Los Angeles activity score (UCLA).

Abbreviations: TKA, total knee arthroplasty; UKA, unicompartmental knee arthroplasty.

Radiographic evaluation of unrevised implants at final follow‐up did not reveal relevant signs of component loosening, implant failure or progressive osteoarthritis requiring further surgical intervention.

## DISCUSSION

The most important finding of the present study is that conversion of medial UKA to TKA was associated with a lower risk of re‐revision compared to UKA‐to‐UKA revision at mid‐term follow‐up. At 10 years, UKA‐to‐TKA revisions demonstrated higher implant survival, while patient‐reported outcomes were comparable between the two revision strategies. Although UKA‐to‐UKA revision represents a less invasive option that may preserve bone stock and native joint structures, the present findings suggest that this strategy may be associated with a higher risk of subsequent failure in a heterogeneous aseptic revision cohort. Nevertheless, UKA‐to‐UKA revision may remain a reasonable treatment option in selected cases with clearly identifiable and surgically addressable failure mechanisms.

Data on re‐revision surgery following conversion of medial UKA to TKA remain limited, although contemporary evidence is steadily emerging. Most available studies report short‐ to mid‐term outcomes. Forlenza et al. and Restrepo et al. observed favourable short‐term results, with 2‐year implant survivorship rates of 93.4% and 96%, respectively [7, 31]. Lombardi et al. reported a re‐revision rate of 4.1% following UKA‐to‐TKA conversion at a mean follow‐up of 6.1 years [21]. Similarly, Ibach et al. demonstrated an 8‐year survivorship free from re‐revision of 94% [13].

Longer‐term data remain scarce. Nwankwo et al. reported a 10‐year implant survival of 95.6% following TKA conversion for aseptic UKA failure [28], whereas Pumford et al. reported a 10‐year survival free from re‐revision of 89% [30]. In contrast, registry‐based studies have reported lower 10‐year implant survival rates of approximately 82%–83% following conversion of UKA to TKA [6, 19]. These discrepancies may reflect differences in patient selection, implant designs, surgical techniques and institutional case volumes.

In the present study, the observed 10‐year implant survival of 95.3% following UKA‐to‐TKA conversion is consistent with previously reported outcomes from high‐volume institutions. This finding suggests that favourable long‐term implant survivorship can be achieved when revision procedures are performed in specialised centres with appropriate expertise.

Evidence on UKA‐to‐UKA revisions remains even more limited. Available studies generally suggest higher failure rates compared with conversion to TKA [4, 22, 31]. Restrepo et al. reported a sixfold increased risk of failure for UKA‐to‐UKA revisions, although a substantial proportion of procedures in that cohort were performed for acute PJI which represented 71% of cases [31]. Because infection represents a distinct clinical entity with different treatment strategies and outcomes and PJI is a known dominant risk factor for re‐revision [12, 16], the present study focused exclusively on aseptic revision cases. Even within this aseptic cohort, UKA‐to‐UKA revision was associated with a higher risk of re‐revision compared with UKA‐to‐TKA conversion. However, revision indications differed substantially between groups, and UKA‐to‐UKA procedures were frequently performed for mechanical problems such as bearing‐related complications.

In addition, the present analysis suggests that outcomes after UKA‐to‐UKA revision may vary depending on the underlying failure mechanism. Revisions performed for mechanical complications or unexplained pain were associated with higher re‐revision rates, whereas revisions performed for bearing‐related problems, aseptic loosening, or periprosthetic fracture demonstrated comparatively favourable outcomes. These observations highlight the importance of careful failure analysis when planning revision surgery. However, given the limited number of events and the exploratory nature of the subgroup analyses, these findings should be interpreted cautiously.

Functional outcomes following revision of UKA have been reported inconsistently. Most available data concern conversion of UKA to TKA, with some studies suggesting outcomes approaching those of primary TKA in selected cases, while others report inferior results compared with primary procedures [2, 9, 13, 19, 23, 30, 32]. In contrast, functional outcomes after UKA‐to‐UKA revision have rarely been reported.

Based on experience from primary knee arthroplasty, preservation of a unicompartmental implant might be expected to result in superior functional outcomes compared with conversion to TKA [37]. However, this assumption was not confirmed in the present cohort. PROMs were comparable between revision strategies, although ROM was significantly greater after UKA‐to‐UKA revision. While the clinical relevance of this difference may be limited, improved ROM may still be relevant for certain patients, particularly for activities of daily living [24, 25, 33].

Patient‐acceptable symptom state (PASS) thresholds are well established for primary UKA but not for revision procedures [8, 11, 34]. Applying a PASS threshold of 30 derived from primary TKA cohorts [14], 86% of UKA‐to‐UKA and 79% of UKA‐to‐TKA patients in this study would meet the PASS. These findings suggest that satisfactory patient‐perceived outcomes can still be achieved in many patients following revision surgery.

Several limitations of this study should be acknowledged. First, the retrospective design introduces the possibility of selection bias, as the choice between UKA‐to‐UKA revision and conversion to TKA was based on surgeon judgement rather than predefined criteria. In addition, revision indications differed between groups, particularly with respect to progression of lateral osteoarthritis, which occurred exclusively in the UKA‐to‐TKA cohort. Second, the number of re‐revision events was relatively small, limiting the statistical power of subgroup and regression analyses. Third, procedures were performed by multiple surgeons over a long study period, which may have introduced variability in surgical technique and perioperative management. Moreover, implant‐related technical factors such as constraint level, fixation strategy and management of bone loss were not analysed in detail and may have influenced revision outcomes. Furthermore, PROM data were not available for all patients, which may introduce attrition bias. Finally, the single‐centre design may limit generalisability of the results compared with large registry‐based studies.

Despite these limitations, the present study provides clinically relevant insights into revision strategies following medial UKA. The cohort represents one of the larger single‐centre series focusing specifically on aseptic revision cases and includes both UKA‐to‐UKA and UKA‐to‐TKA procedures. The relatively long follow‐up period and the use of validated PROMs further strengthen the analysis.

## CONCLUSIONS

Conversion of medial UKA to TKA was associated with lower re‐revision rates compared with UKA‐to‐UKA revision at mid‐term follow‐up, while patient‐reported outcomes were comparable between revision strategies. UKA‐to‐UKA revision may remain a reasonable option in selected cases with clearly identifiable and surgically addressable failure mechanisms. These findings support an individualised revision strategy that considers the underlying failure mechanism and patient‐specific factors.

## AUTHOR CONTRIBUTIONS

**Kevin‐Arno Koch**: Conceptualisation and methodology; investigation; formal analysis and visualisation; writing—original draft preparation; supervision. **Jakob Freytag**: Investigation; formal analysis and visualisation. **Mustafa Hariri**: Investigation; writing—review and editing; supervision. **Raphael Trefzer**: Writing—review and editing. **Paul Mick**: Writing—review and editing. **Andre Lunz**: Writing—review and editing. **Tilman Walker**: Conceptualisation and methodology; writing—review and editing; supervision. **Johannes Weishorn**: Conceptualisation and methodology; investigation; writing—review and editing; supervision.

## FUNDING INFORMATION

None.

## CONFLICT OF INTEREST STATEMENT

The authors declare no conflicts of interest.

## ETHICS STATEMENT

The current study was approved by the Ethics Commission of the Medical Centre, University of Heidelberg (S‐944/2021). Written informed consent was obtained from every patient before inclusion.

## Data Availability

The data will be available upon reasonable request.
